# Histological Differentiated/Undifferentiated Mixed Type Should Not Be Considered as a Non-Curative Factor of Endoscopic Resection for Patients With Early Gastric Cancer

**DOI:** 10.3389/fonc.2020.01743

**Published:** 2020-09-03

**Authors:** Xiaolong Tang, Mengjun Zhang, Qingsi He, Guorui Sun, Chao Wang, Peng Gao, Hui Qu

**Affiliations:** ^1^Department of General Surgery, Qilu Hospital of Shandong University, Jinan, China; ^2^Department of General Surgery, Lanling People’s Hospital, Linyi, China; ^3^Department of Pathology, Qilu Hospital of Shandong University, Jinan, China

**Keywords:** early gastric cancer, endoscopic resection, histological type, gastrectomy, prognosis

## Abstract

**Background:**

Histological differentiated/undifferentiated mixed-type adenocarcinomas are frequently found in patients with early gastric cancer (EGC). Yet it is unclear whether these mixed-type adenocarcinomas can be treated by endoscopic resection (ER) in EGC patients.

**Aims:**

To evaluate the lymph node metastasis (LNM) rate and long-term outcomes in mixed-type EGC patients and assess the feasibility of ER in these patients.

**Methods:**

Clinicopathological features, risk factors of LNM, and overall survival (OS) and progression-free survival (PFS) rates of EGC patients were analyzed according to different histological types.

**Results:**

Patients with mixed-type EGC had higher LNM rates than patients with non-mixed-type EGC (11.4 vs. 6.2%, *P* = 0.044). In the multivariate analysis, larger tumor diameter, presence of an ulcer, submucosal invasion, histological undifferentiated type, histological mixed type, and lymphovascular invasion resulted as independent risk factors for LNM in EGC patients (all *P* < 0.05). The LNM rate in mixed-type patients who met the Japanese ER criteria was 3.3%, including fulfilling the absolute criteria 0%. The 5-year OS and PFS rates in mixed-type patients were 94.59 and 91.47%, respectively. There was no statistical significance in the OS (*P* = 0.870) and PFS (*P* = 0.705) between mixed-type and non-mixed-type EGC patients fulfilling the Japanese ER criteria.

**Conclusion:**

Histological differentiated/undifferentiated mixed type in EGC patients meeting the Japanese absolute criteria for ER are associated with low risk of LNM and favorable prognosis, and thus, it should not be considered as a non-curative factor for ER.

## Introduction

Early gastric cancer (EGC) can be histologically classified into differentiated and undifferentiated types (1); the former contains well or moderately differentiated papillary/tubular adenocarcinoma. In contrast, the latter contains poorly differentiated/signet-ring cell/mucinous adenocarcinoma. In addition to these histological types, the differentiated/undifferentiated mixed-type adenocarcinomas, which refer to the intermingled histopathology of differentiated and undifferentiated adenocarcinoma components, are frequently observed in EGC patients (2). The histological types are considered when determining different treatment strategies for EGC patients. For example, for undifferentiated-type EGC, radical gastrectomy with lymph node dissection is generally selected rather than endoscopic submucosal dissection (ER) because this type is believed to be associated with lymph node metastasis (LNM) (3). However, the LNM rates in EGC patients with differentiated/undifferentiated mixed type still remain controversial. Huh et al. (4) reported that, in EGC patients, adenocarcinoma mixed with signet-ring cell histological components showed more aggressive behavior than other histology and was an independent risk factor for LNM (*P* < 0.001). Takizawa et al. (5) retrospectively analyzed the LNM rates of different histological types in EGC patients, identifying no statistical significance in LNM rates among mixed type and purely undifferentiated/differentiated type (5.9 vs. 5.1/2.4%, *P* = 0.124).

Although previous studies focused on the LNM in EGC patients, the LNM rates in patients with mixed-type EGC meeting the ER criteria still remain unclear (6). Moreover, data on the long-term outcomes of mixed-type EGC are scarce, and it is unclear whether mixed type is a non-curative factor for EGC patients fulfilling the ER criteria (7). Therefore, the aim of this study was to analyze the LNM rates and long-term outcomes in patients with mixed-type EGC meeting the criteria for ER and evaluate the feasibility of ER in these patients.

## Materials and Methods

### Patients

All 1174 EGC cases registered at Qilu Hospital at Shandong University between February 2003 and December 2018 were prospectively reviewed. The inclusion criteria were as follows: (1) patients underwent radical gastrectomy with D2 lymph node dissection, (2) patients with complete clinical and pathological data, and (3) patients had a postoperative histological diagnosis that confirmed adenocarcinoma. The exclusion criteria were as follows: (1) synchronous multiple gastric tumors, (2) patients with a history of gastrectomy before surgery, (3) patients who died of postoperative complications, (4) patients with less than 16 retrieved lymph nodes, and (5) patients lost to follow-up. Preoperative examinations, including gastroscopy, endoscopic ultrasound, and abdominal computed tomography, were performed in all patients (8). Postoperative follow-up examinations were scheduled at 6-month intervals during the first year and once a year thereafter. Follow-up data were prospectively collected and updated until February 2020. The informed consent was waived due to the retrospective nature of the study. This study was approved by the Ethics Committee Board of Qilu Hospital of Shandong University, China.

### Data Collection

The collected data included the patient’s age, gender, tumor diameter, tumor location of the stomach, surgical type (subtotal or total gastrectomy), macroscopic appearance (elevated, flat, depressed), presence of an ulcer, depth of invasion (mucosal or submucosal), number of dissected lymph nodes, LNM, pathological tumor stage (pTNM), lymphovascular invasion, perineural invasion, and histological differentiation (purely differentiated, purely undifferentiated, differentiated/undifferentiated mixed type). The intervals between sections of the primary lesion were 2–3 mm. Continuous multiple sectioning was used at 2-mm intervals in the preparation of lymph node sections. The D2-40 was used in lymphatic vessel stains, and CD31/CD34 was used in venous stains. Immunostaining (CK/CK-19) was used for the evaluation of lymph node metastasis. An isolated tumor cell or micrometastasis in the lymph node was defined as metastasis. Lymphovascular invasion (LVI) was defined as carcinoma cells present within a definite, endothelial-lined space (the lymphatic and/or blood vessels). Lymphatic vessels (ly) were not differentiated from the blood vessels (v) in LVI-positive patients. Pathologists reviewed mixed-type EGC pathological slides to evaluate the predominantly differentiated/undifferentiated components needed to meet the ER criteria (9). Pathological tumor stages were classified according to the American Joint Committee on Cancer staging system (7th edition).

### ER Criteria

To evaluate the feasibility of ER in mixed-type EGC patients, LNM rates were compared for each criterion (absolute or expanded criteria) of the 2018 Japanese ER guidelines (5th edition) (10). Based on the postoperative pathological reports, all EGC cases were classified into the following ER criteria: absolute criterion (intramucosal, non-ulcerated, predominantly histologically differentiated, tumor diameter ≤2 cm) or expanded criterion (criterion #1: intramucosal, non-ulcerated, histologically differentiated, tumor diameter >2 cm; criterion #2: intramucosal, ulcerated, histologically differentiated, tumor diameter ≤3 cm; criterion #3: intramucosal, non-ulcerated, histologically undifferentiated, tumor diameter ≤2 cm; criterion #4: submucosal invasion <500 μm [SM1], histologically differentiated, tumor diameter ≤3 cm) (11).

### Statistical Analysis

Continuous variables were presented as medians (ranges). Differences in distributions of baseline characteristics among participants with mixed-type and non-mixed-type cancers were analyzed with the use of Pearson’s chi-square test for categorical variables and the Mann–Whitney test for ordinal or continuous variables with a non-normal distribution. The Kaplan–Meier method was used to calculate OS curves based on the length of time between primary surgical treatment and final follow-up or death and PFS curves based on the length of time between primary surgical treatment and final follow-up, death, recurrence, or metastasis. The log-rank test was used to assess statistical differences between curves. The logistic regression model was used to identify the independent factors associated with LNM in EGC patients (12). *P* < 0.05 was considered statistically significant. The statistical analysis was performed using SAS 9.4 (SAS Institute Inc., Kerry, United States).

## Results

### Comparison of Clinicopathologic Features Among Mixed, Differentiated, and Undifferentiated Type in EGC Patients

A total of 853 EGC cases were enrolled in this study, including 105 (12.3%) mixed type, 405 (47.5%) differentiated type, and 343 (40.2%) undifferentiated type cases. The comparison of clinicopathologic features among patients with three different histological types are shown in Table 1. Mixed-type EGC tumors were associated with younger age (*P* = 0.000), female gender (*P* = 0.000), larger tumor diameter (*P* = 0.020), more located at the body of the stomach (*P* = 0.000), more flat macroscopic appearance (*P* = 0.000), more positive LNM (*P* = 0.001), higher pN stage (*P* = 0.004), and higher pTNM stage (*P* = 0.004) compared to non-mixed-type (differentiated-type or undifferentiated-type) EGC tumors. There was no significant difference among these three groups in relation to surgical type, presence of an ulcer, depth of invasion (the pT stage), number of dissected lymph nodes, lymphovascular invasion, perineural invasion or follow-up periods (all *P* > 0.05).

**TABLE 1:** Comparison of clinicopathologic features among three different histological types in EGC patients.

Variables	Mixed type (*n* = 105)	Differentiated type (*n* = 405)	Undifferentiated type (*n* = 343)	χ^2^/F	*p*-value
**Age (years)**				34.78	**0.000**
≤60	69(65.71)	168(41.48)*	206 (60.06)		
>60	36 (34.29)	237 (58.52)	137 (39.94)		
Gender				33.44	**0.000**
Male	65 (61.90)	321(79.26)*	259(75.51)*		
Female	40 (38.10)	84 (20.74)	84 (24.49)		
**Tumor diameter (cm)**				7.83	**0.020**
≤2.0	52 (49.52)	259(63.95)*	200 (58.31)		
>2.0	53 (50.48)	146 (36.05)	143 (41.69)		
**Tumor location**				65.25	**0.000**
Cardiac and bottom	8 (7.62)	61 (15.06)	95(27.70)*		
Body	44 (41.90)	70 (17.28)	63 (18.37)		
Antrum	53 (50.48)	274 (67.66)	185 (53.93)		
**Surgical type**				1.14	0.564
Subtotal gastrectomy	93 (88.57)	366 (90.37)	315 (91.84)		
Total gastrectomy	12 (11.43)	39 (9.63)	28 (8.16)		
**Macroscopic appearance**				22.77	**0.000**
Elevated	40 (38.10)	230(56.79)*	164 (47.81)		
Flat	44 (41.90)	96 (23.70)	86 (25.07)		
Depressed	21 (20.00)	79 (19.51)	93 (27.11)		
**Presence of ulcer**				4.14	0.126
Yes	15 (14.29)	41 (10.12)	51 (14.87)		
No	90 (85.71)	364 (89.88)	292 (85.13)		
**Depth of invasion (pT stage)**				4.58	0.101
Mucosa (pT1a)	79 (75.24)	261 (64.44)	223 (65.01)		
Submucosa (pT1b)	26 (24.76)	144 (35.56)	120 (34.99)		
No. of dissected lymph nodes [mean ± SD]	23.11 ± 8.32	22.35 ± 5.56	22.88 ± 5.85	1.04	0.354
**Lymph node metastasis**				14.16	**0.001**
Positive	12 (11.43)	14(3.46)*	32 (9.33)		
Negative	93 (88.57)	391 (96.54)	311 (90.67)		
**pN stage**				18.96	**0.004**
pN0	93 (88.57)	391(96.54)*	311 (90.67)		
pN1	8 (7.62)	7 (1.73)	12 (3.50)		
pN2	3 (2.86)	5 (1.23)	14 (4.08)		
pN3a	1 (0.95)	2 (0.49)	6 (1.75)		
**pTNM stage**				18.96	**0.004**
1a	93 (88.57)	391(96.54)*	311 (90.67)		
1b	8 (7.62)	7 (1.73)	12 (3.50)		
2a	3 (2.86)	5 (1.23)	14 (4.08)		
2b	1 (0.95)	2 (0.49)	6 (1.75)		
**Lymphovascular invasion**				2.53	0.282
Positive	8 (7.62)	18 (4.44)	23 (6.71)		
Negative	97 (92.38)	387 (95.56)	320 (93.29)		
**Perineural invasion**				4.16	0.125
Positive	1 (0.95)	4 (0.99)	1 (0.29)		
Negative	104 (99.05)	401 (99.01)	342 (99.71)		
Follow-up [months, median (range)]	64(43−195)	62(32−198)	64(45−204)	1.00	0.369

*Represents a statistical difference between the mixed-type group and the differentiated-/undifferentiated-type group. EGC, early gastric cancer; pT: pathological tumor; pN, pathological lymph node; pTNM, pathological tumor-node-metastasis. All *P* values < 0.05 were marked in bold print.

### Univariate and Multivariate Analysis for Risk Factors of LNM in EGC Patients

Among the 853 EGC patients enrolled, 58 (6.80%) had LNM. The LNM rate in patients with mixed type was 11.43% (12/105), and the LNM rate in patients with non-mixed type was 6.15% (46/748). In the univariate analysis, larger tumor diameter (*P* = 0.000), presence of ulcer (*P* = 0.002), depth of invasion (*P* = 0.003), histological type (*P* = 0.001), and lymphovascular invasion (*P* = 0.000) were associated with LNM in EGC patients. According to the multivariate analysis, diameter >2.0 cm [odds ratio (OR) 3.59, 95% confidence interval (CI) 1.93–6.69, *P* < 0.001], presence of ulcer (OR 2.83, 95% CI 1.44–5.55, *P* = 0.003), submucosal invasion (OR 1.86, 95% CI 1.04–3.34, *P* = 0.037), undifferentiated-type histology (OR 2.77, 95% CI 1.40–5.47, *P* = 0.004), mixed-type histology (OR 3.55, 95% CI 1.50–8.39, *P* = 0.004), and lymphovascular invasion (OR 7.86, 95% CI 3.78–16.33, *P* < 0.001) resulted as independent risk factors for LNM in EGC patients. The details of the univariate and multivariate analysis are shown in Table 2.

**TABLE 2:** Univariate and multivariate logistic analysis of factors associated with lymph node metastasis in EGC patients.

Variables	No. of cases	LNM cases (%)	Univariate	Multivariate	
				
			*p*-value	OR (95% CI)	*p*-value
**Age (years)**			0.061		
≤60	443	37 (8.35)			
>60	410	21 (5.12)			
**Gender**			0.124		
Male	645	39 (6.05)			
Female	208	19 (9.13)			
**Tumor diameter (cm)**			**0.000**		
≤2.0	511	16 (3.13)		1.00	
>2.0	342	42 (12.28)		3.59 (1.93–6.69)	**<0.001**
**Tumor location**			0.144		
Cardiac and bottom	164	7 (4.27)			
Body	177	17 (9.6)			
Antrum	512	34 (6.64)			
**Surgical type**			0.768		
Subtotal gastrectomy	774	52 (6.72)			
Total gastrectomy	79	6 (7.59)			
Macroscopic appearance			0.827		
Elevated	434	28 (6.45)			
Flat	226	15 (6.64)			
Depressed	193	15 (7.77)			
**Presence of ulcer**			**0.002**		
No	746	43 (5.76)		1.00	
Yes	107	15 (14.02)		2.83 (1.44–5.55)	**0.003**
**Depth of invasion (pT stage)**			**0.003**		
Mucosa (pT1a)	563	28 (4.97)		1.00	
Submucosa (pT1b)	290	30 (10.34)		1.86 (1.04–3.34)	**0.037**
**Tumor differentiation**			**0.001**		
Differentiated-type	405	14 (3.46)		1.00	
Undifferentiated-type	343	32 (9.33)		2.77 (1.40–5.47)	**0.004**
Mixed-type	105	12 (11.43)		3.55 (1.50–8.39)	**0.004**
**Lymphovascular invasion**			**0.000**		
Negative	804	43 (5.35)		1.00	
Positive	49	15 (30.61)		7.86 (3.78–16.33)	**<0.001**
**Perineural invasion**			0.507		
Negative	847	58 (6.85)			
Positive	6	0 (0)			

*EGC, early gastric cancer; OR, odds ratio; CI, confidence interval; LNM, lymph node metastasis; pT, pathological tumor. All *P* values < 0.05 were marked in bold print.*

### LNM Rate of Mixed-Type ECG Patients According to the Japanese Absolute or Expanded ER Criteria

To investigate whether patients with mixed-type EGC should undergo ER, we reviewed the LNM rate in mixed-type EGC patients according to the 2018 Japanese ER criteria (10). Among the 853 EGC patients, 555 (65.1%) met the criteria for ER, including 215 (25.2%) who fulfilled the absolute criteria and 340 (39.9%) who fulfilled the expanded criteria. Among the 105 patients with mixed-type EGC, 64 (60.9%) were predominantly histologically differentiated, and 41 (39.1%) predominantly undifferentiated. Among these mixed-type EGC patients, 60 (57.1%) met the absolute or expanded ER criteria. The LNM rates among all EGC and mixed-type patients meeting the ER criteria were 3.8% (21/555) and 3.3% (2/60), respectively. The mixed-type EGC patients who fulfilled the absolute ER criteria had no LNM (0/28), and those who fulfilled the expanded criteria #1, #2, #3, or #4 had LNM rates of 8.3% (1/12), 12.5% (1/8), 0% (0/8), and 0% (0/4), respectively. As shown in Table 3, mixed-type EGC patients with intramucosal tumors without ulcerative findings and diameter ≤2 cm did not have LNM regardless of histologically differentiated (absolute criteria) or undifferentiated (expanded criteria #3) status. Moreover, no lymph node metastasis was found in the LVI negative (ly0 and v0) patients.

**TABLE 3:** Number of lymph node metastases in all cases and mixed-type EGC patients fulfilling different Japanese endoscopic resection criteria.

Variables	Absolute criterion	Expanded criterion #1	Expanded criterion #2	Expanded criterion #3	Expanded criterion #4	Total
**All EGC patients**						
No. of cases	215	95	28	127	90	555
LNM cases [n (%)]	3 (1.4)	2 (2.1)	1 (3.6)	9 (7.1)	6 (6.7)	21 (3.8)
**Mixed-type EGC patients**						
No. of cases	28	12	8	8	4	60
LNM cases [n (%)]	0 (0)	1 (8.3)	1 (12.5)	0 (0)	0 (0)	2 (3.3)

*EGC, early gastric cancer; LNM, lymph node metastasis.*

### Long-Term Outcomes of Mixed-Type ECG Patients Fulfilling the ER Criteria

**FIGURE 1:**
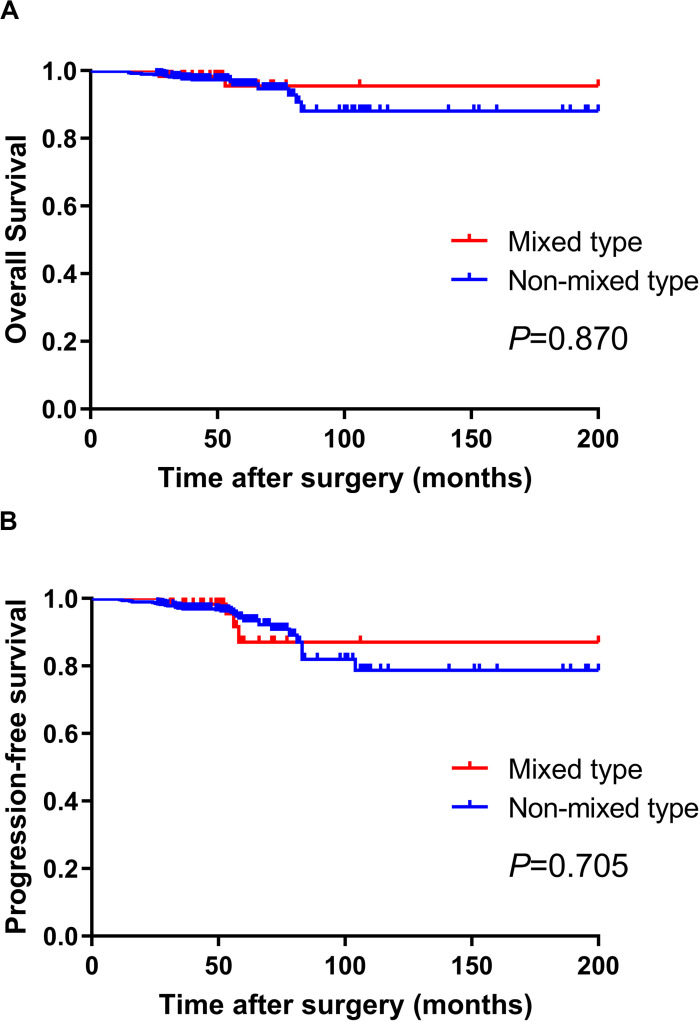
There was no significant difference in OS **(A)** and PFS **(B)** between mixed-type and non-mixed-type EGC patients who met the Japanese ER criteria (*P* > 0.05).

**FIGURE 2:**
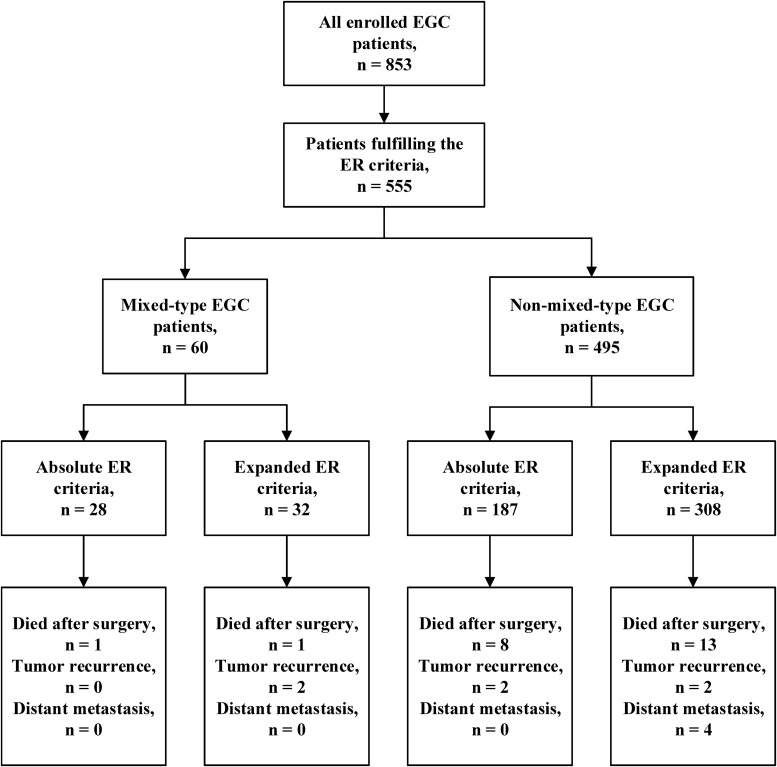
Flowchart for long-term outcomes in EGC patients who met the Japanese ER criteria.

We further analyzed the prognosis of EGC patients who met the criteria for ER (absolute or expanded) according to different histological types (13). The median follow-up period for all EGC patients was 64 (range 32–204) months. Among the 60 mixed-type EGC patients who met the criteria for ER, 2 (3.3%) patients died after surgery, and 2 (3.3%) patients experienced tumor recurrence in the remnant stomach. There was no gastric cancer–related death or distant metastasis occurring during the follow-up period. The 5-year OS and PFS rates in mixed-type patients were 94.59 and 91.47%, respectively. During the same follow-up period, among the 495 non-mixed-type patients fulfilling criteria for ER, 21 (4.2%) patients died, including 4 gastric cancer–related deaths. Six (1.2%) patients had tumor recurrence, and 4 (0.8%) patients had distant metastasis. The 5-year OS and PFS rates of non-mixed-type patients were 94.81 and 93.06%, respectively. The Kaplan–Meier analysis shows that there was no significant difference in both OS (*P* = 0.870) and PFS (*P* = 0.705) between the two groups. The survival curves of OS and PFS are shown in Figure 1. A flowchart with long-term outcomes of mixed-type and non-mixed-type EGC patients fulfilling the ER criteria is shown in Figure 2.

## Discussion

Following the fast development of endoscopic technology, ER has become an important option for patients with EGC. According to the latest 2018 Japanese gastric cancer treatment guidelines (5th edition), ER criteria are mainly based on histological types, depth of invasion (pT stage), ulcerative findings, and tumor diameter, which make the histological types very important for EGC patients (10). The Japanese guidelines have specified the criteria for ER in patients with histologically differentiated or undifferentiated adenocarcinomas (14). Well or moderately differentiated EGC usually implies low risk of LNM and curative ER (11, 15). Interestingly, many studies have reported that patients with pure undifferentiated histological types also have a favorable prognosis (3, 16–18). According to these results, the foundation for ER in patients with histologically differentiated or undifferentiated types EGC was established (19). However, the feasibility of ER in patients with histologically differentiated/undifferentiated mixed-type adenocarcinomas still remained unclear.

In this study, we compared the clinicopathological features of EGC patients with mixed type and non-mixed type. The mixed-type tumors showed more aggressive behaviors, such as younger age, larger tumor diameter, more positive LNM, higher pN stage, and higher pTNM stage (20). Moreover, in the univariate and multivariate logistic analysis, histological mixed type resulted as an independent risk factor for LNM in patients with EGC. This was consistent with previous studies, which also found that the mixed type was more associated with younger age, positive LNM, lymphovascular invasion, and depressed type than other histological types (21). The locally aggressive characteristics in mixed-type EGC may be explained by the increased expression of proteins, such as Ki-67, EMMPRIN, and VEGF, which are involved in the angiogenetic process and cell proliferation in mixed-type EGC (22). Another study suggests that mixed-type gastric carcinomas frequently show CpG island hypermethylation (23).

As is well known, treatment for EGC mainly depends on the risk of LNM and long-term outcomes. Previous studies have shown that the LNM rate in EGC patients ranges from 5% to 18% according to the proportion of submucosa invasion (24). However, the mixed-type EGC shows more aggressive behaviors with a higher LNM rate than other histological types. Huh et al. reviewed 156 mixed-type patients and reported an LNM of 19.2% in mixed-type EGC (4). In our study, the LNM of mixed-type EGC was 11.4%, which was significantly higher than in patients with non-mixed type (6.2%). These findings suggest that the overall LNM rates in mixed-type EGC may not be suitable for ER (25). To further clarify this doubt, we calculated the LNM rate in mixed-type EGC patients meeting the Japanese criteria for ER. The results show that, under the absolute criteria, the LNM in mixed-type EGC was 0% regardless of the predominant histological proportion, which means that EGC patients with mixed-type histology, intramucosal invasion, no ulcerative findings, and diameter ≤2 cm have a very low risk of LNM (26).

As for mixed-type EGC patients meeting the expanded criteria, the LNM rates were 8.3% (1/12, criteria #1), 12.5% (1/8, criteria #2), 0% (0/8, criteria #3), and 0% (0/4, criteria #4), respectively. The LNM rate in patients meeting expanded criteria #1 and #2 appears high, which may be caused by the small number of patients in each group. Moreover, the low LNM rates (0%) among patients meeting expanded criteria #3 (8 cases) and #4 (only 4 cases) may also be caused by the same reason. The 2018 Japanese gastric cancer guidelines newly defined the ER curability (eCura) criteria (27). According to these criteria, EGC patients who met the absolute or expanded criteria for ER received en bloc ER with negative horizontal/vertical margins and had no lymphovascular infiltration should be regarded as suitable candidates for endoscopic treatment (28). Observation and follow-up with necessary examinations should be enough for these patients (29). During our follow-up period, mixed-type EGC patients fulfilling the ER criteria had a 5-year OS rate of 94.59% and a PFS rate of 91.47%. Among 60 patients, only 2 (3.3%) patients died after surgery, and 2 (3.3%) experienced tumor recurrence in the remnant stomach. There was no gastric cancer–related death during the follow-up period. Moreover, the 5-year OS and PFS in mixed-type patients were similar to that of non-mixed-type patients. Although mixed type is a clear risk factor for LNM in EGC patients, the rate of LNM seems to be negligible under certain conditions (30). This implies the mixed-type EGC patients meeting the absolute ER criteria should not be considered as non-curative for ER in EGC patients. For these patients, additional surgery after the ER may not be necessary (31).

## Conclusion

For EGC patients meeting the Japanese criteria for ER, the mixed type indicates a high risk of LNM; however, in patients fulfilling the absolute or expanded ER criteria, mixed type may indicate a low risk of LNM and favorable prognosis. Mixed type patients who met the absolute ER criteria did not have LNM, and there was no significant difference in the OS and PFS between mixed-type and non-mixed-type EGC patients. Mixed-type histology in EGC patients meeting the Japanese absolute criteria for ER should not be considered as non-curative for endoscopic treatment. For patients meeting the expanded ER criteria, this case series is still limited, and further data are needed.

## Data Availability Statement

The raw data supporting the conclusions of this article will be made available by the authors, without undue reservation.

## Ethics Statement

The studies involving human participants were reviewed and approved by the Ethics Committee of Qilu Hospital of Shandong University. The patients/participants provided their written informed consent to participate in this study. Written informed consent was obtained from the individual(s) for the publication of any potentially identifiable images or data included in this manuscript.

## Author Contributions

HQ, XT, and PG designed this study. XT and MZ contributed in drafting the manuscript. HQ, QH, and GS critically revised the manuscript for important intellectual content. XT, MZ, and CW collected and analyzed the data. All the authors approved the final manuscript submitted. Each author participated sufficiently in the work to take public responsibility for appropriate portions of the content and agreed to be accountable for all aspects of the work in ensuring that questions related to the accuracy or integrity of any part of the work are appropriately investigated and resolved.

## Conflict of Interest

The authors declare that the research was conducted in the absence of any commercial or financial relationships that could be construed as a potential conflict of interest.
